# Long-term outcomes after video-assisted thoracic surgery (VATS) lobectomy versus lobectomy via open thoracotomy for clinical stage IA non-small cell lung cancer

**DOI:** 10.1186/1749-8090-9-88

**Published:** 2014-05-17

**Authors:** Mitsunori Higuchi, Hiroshi Yaginuma, Atsushi Yonechi, Ryuzo Kanno, Akio Ohishi, Hiroyuki Suzuki, Mitsukazu Gotoh

**Affiliations:** 1 Department of Thoracic Surgery, Fukushima Red Cross Hospital, Fukushima, Japan; 2 Department of Thoracic Surgery, Fukushima Medical University School of Medicine, 1-Hikarigaoka, Fukushima 960-1295, Japan

**Keywords:** Clinical stage IA, Lobectomy, Open thoracotomy, Prognosis, Video-assisted thoracic surgery (VATS)

## Abstract

**Background:**

Video-assisted thoracic surgery (VATS) lobectomy is a standard treatment for lung cancer. This study retrospectively compared long-term outcomes after VATS lobectomy versus lobectomy via open thoracotomy for clinical stage IA non-small cell lung cancer (NSCLC).

**Methods:**

From July 2002 to June 2012, 160 patients were diagnosed with clinical stage IA NSCLC and underwent lobectomy. Of these, 114 underwent VATS lobectomy and 46 underwent lobectomy via open thoracotomy.

**Results:**

The 5-year disease-free survival (DFS) rate was 88.0% in the VATS group and 77.1% in the thoracotomy group for clinical stage IA NSCLC (*p* = 0.1504), and 91.5% in the VATS group and 93.8% in the thoracotomy group for pathological stage IA NSCLC (*p* = 0.2662). The 5-year overall survival (OS) rate was 94.1% in the VATS group and 81.8% in the thoracotomy group for clinical stage IA NSCLC (*p* = 0.0268), and 94.8% in the VATS group and 96.2% in the thoracotomy group for pathological stage IA NSCLC (*p* = 0.5545). The rate of accurate preoperative staging was 71.9% in the VATS group and 56.5% in the thoracotomy group (*p* = 0.2611). Inconsistencies between the clinical and pathological stages were mainly related to tumor size, nodal status, and pleural invasion. Local recurrence occurred for one lesion in the VATS group and six lesions (five patients) in the thoracotomy group (*p* = 0.0495).

**Conclusions:**

The DFS and OS were not inferior after VATS compared with thoracotomy. Local control was significantly better after VATS than after thoracotomy. Preoperative staging lacked sufficient accuracy.

## Background

Lung cancer is the leading cause of cancer-related deaths worldwide [1]. Surgical resection of lung cancer in patients without metastasis to other organs may provide a long-term survival benefit compared with nonsurgical therapies. Robert McKenna first described video-assisted thoracic surgery (VATS) lobectomy in 1994 [2]. The findings of many studies have established with reasonable certainty that VATS lobectomy is associated with less postoperative pain, less postoperative morbidity, and shorter hospital stay than lobectomy via open thoracotomy [3-6]. Several studies also reported that VATS lobectomy is feasible and safe [4,5]. We started performing VATS lobectomy in selected patients with stage IA non-small cell lung cancer (NSCLC) in 2004. After an initial learning curve period, we also started performing this procedure in patients with stage IB NSCLC, as we believed that the outcomes would not be inferior to those after open thoracotomy. This study compared the long-term survival outcomes after VATS lobectomy versus lobectomy via open thoracotomy for clinical stage IA NSCLC, and evaluated the current concerns in these groups of patients.

## Methods

### Patient characteristics

This retrospective study included 160 consecutive eligible patients treated in the Department of Thoracic Surgery at Fukushima Red Cross Hospital from July 2002 to June 2012. The inclusion criteria were: VATS lobectomy or lobectomy via open thoracotomy, with curative intent and without preoperative induction chemotherapy or radiotherapy; and a definitive postoperative diagnosis of stage IA NSCLC according to the latest revision of the international system for staging lung cancer [7]. The study included 89 females and 68 males. The hospital and office records of each patient were reviewed and demographic and clinical data were recorded, including age, sex, pathology, clinical stage, and other clinicopathological factors. The characteristics of the patients are shown in Table 1. The protocol was conducted in accordance with the Declaration of Helsinki and Good Clinical Practice guidelines. This study was approved by the Ethical Committee of Fukushima Red Cross Hospital.

**Table 1:** Patient characteristics

	**No. of patiens (%)**	**VATS (n = 114)**	**Thoracotomy (n = 46)**	**p**
Gender				0.002
Male	84(52.5)	51	33	
Female	76(47.5)	63	13	
Age				0.1001
≤70	85(53.1)	58	27	
>	75(46.9)	56	19	
Pathology				0.1533
Adenocarcinoma	123(769)	91	31	
Squamous cell ca.	28(17.5)	17	11	
Large cell ca.	3(1.9)	3	0	
Others	6(3.7)	3	3	
Tumor diameter (cm)				0.895
≤3.0	138(86.3)	100	38	
3.0<	22(13.7)	14	8	
pN				0.1706
N0	141(88.1)	103	38	
N1/N2	19(11.9)	11	8	
Pleural involvement				0.2413
Negative	122(76.2)	93	29	
Positive	8(23.8)	21	17	
Lymphovascular involvement				0.0025
Negative	135(84.4)	98	37	
Positive	25(15.6)	16	9	
Vascular involvement				0.0126
Negative	120(75.0)	93	27	
Positive	40(25.0)	21	19	
p-stage				0.2611
IA	108(67.5)	82(71.9)	26(56.5)	
IB	30(18.8)	20	10	
IIA	10(6.3)	6	4	
IIB	3(1.8)	1	2	
IIIA	9(5.6)	5	4	
Recurrence	160(100)	12	10	0.0623
Death	160(100)	6	9	0.005
B1eeding (g)	160(100)	118.1 ± 154.3	321.6 ± 352.4	<0.0001
Surgery time (min.)	160(100)	198.3 ± 50.2	205.1 ± 60.9	0.4662

### Preoperative staging

Preoperative investigations included thoracic and upper abdominal computed tomography (CT) and brain magnetic resonance imaging (MRI) to establish absence of multiple pulmonary lesions and absence of hepatic, adrenal, or brain metastases; and to evaluate hilar and mediastinal lymph node status. Bone scintigraphy was performed if clinically indicated. F18-fluorodeoxyglucose positron emission tomography (FDG-PET) was performed within one month before surgery only when a preoperative histological diagnosis could not be obtained.

### Surgical procedures

All patients underwent curative surgery by VATS lobectomy or lobectomy via open thoracotomy. Patients were placed in the lateral decubitus position with single-lung ventilation. For VATS, a 10-mm, 30° thoracoscope was introduced through the 7^th^ intercostal space in the midaxillary line. A 2-cm incision was made in the 8^th^ intercostal space in the auscultatory triangle. A 5-cm access thoracotomy was usually placed in the 4^th^ or 5^th^ intercostal space in the anterior axillary line for upper/middle or lower lobectomy, respectively. The pulmonary vessels and bronchi were dissected as for open thoracotomy. Branches of the pulmonary artery were ligated with 3–0 Vicryl (Ethicon, Somerville, NJ, USA) or stapled with a linear stapler (Covidien, Mansfield, MA, USA). The endoscopic linear stapler was used to divide pulmonary veins and bronchi, and plication of the fissure. The resected tissue was placed in a plastic specimen bag for retrieval to avoid implantation of tumor cells. Lobectomy via open thoracotomy was performed using a 20cm posterolateral incision sparing the serratus anterior muscle, through the 4^th^ or 5^th^ intercostal space. The vascular and bronchial structures were individually dissected and divided using endoscopic staplers. Complete hilar and mediastinal lymph node dissection was usually performed during both VATS and open thoracotomy. In patients with severe preoperative morbidity or aged >80 years, only hilar dissection and mediastinal lymph node sampling were performed. Resection was considered complete when the resection margins were free of disease. Pathological staging was performed according to the 7^th^ International Staging System for Lung Cancer [7].

### Histopathological examination

Tumors were evaluated by an experienced pathologist, and graded according to the 2004 World Health Organization classification for NSCLC. All specimens were formalin-fixed, paraffin-embedded, and stained with hematoxylin and eosin. Pleural invasion, lymphatic involvement, and vascular involvement were determined by elastica-Masson and hematoxylin and eosin staining.

### Treatment and follow-up

Outpatient follow-up was performed by thoracic surgeons every 2–3 months until 12 months after surgery, then every 6 months until 60 months, and then yearly. Standard follow-up consisted of chest X-ray, laboratory testing including measurement of tumor markers, and clinical examination. Chest CT was performed every 6 months until 2 years, and then yearly. Further CT was only performed if there were suspicious radiological, serological, or clinical findings. Adjuvant chemotherapy was administered to patients with adenocarcinoma if the tumor measured >2.0 cm in diameter, using oral tegafur/uracil (UFT™, Taiho Pharmaceuticals, Tokyo, Japan) for 2 years according to the Japanese standard adjuvant chemotherapy regimen [8]. In patients with stage II or more advanced stage NSCLC, vinorelbine (Navelbine™, Kyowa Hakkou Kirin Co., Ltd, Tokyo, Japan) and cisplatin (Randa™, Nippon Kayaku Co., Ltd, Tokyo, Japan) were administered intravenously for four cycles according to the report of the JBR.10 trial [9,10]. Adjuvant chemotherapy was administered only to patients aged <75 years. The overall survival (OS) was estimated from the date of surgical resection until death of any cause or the date of last follow-up. The disease-free survival (DFS) was estimated from the date of surgical resection until tumor recurrence or death from any cause. Local recurrence was defined as recurrence in the pleural cavity or mediastinal or hilar nodal stations. Distant recurrence was defined as recurrence in the distant organs or the ipsilateral or contralateral lung.

### Statistical analysis

Clinicopathological factors were compared between groups using the two-tailed Pearson’s chi-square test. Survival probabilities were estimated using the Kaplan–Meier method. The significance of differences in disease-free survival (DFS) between groups was tested using the log-rank test. The univariate Cox proportional hazards model was used to quantify the risk of recurrence as a function of sex, age, histology, tumor size, and lobectomy procedure. The multivariate Cox proportional hazards model was used to identify independent predictors of outcome after VATS lobectomy. All statistical analyses were performed using StatView 5.0 (SAS Institute, Cary, NC, USA).

## Results

### Disease-free and overall survival

**Figure 1:**
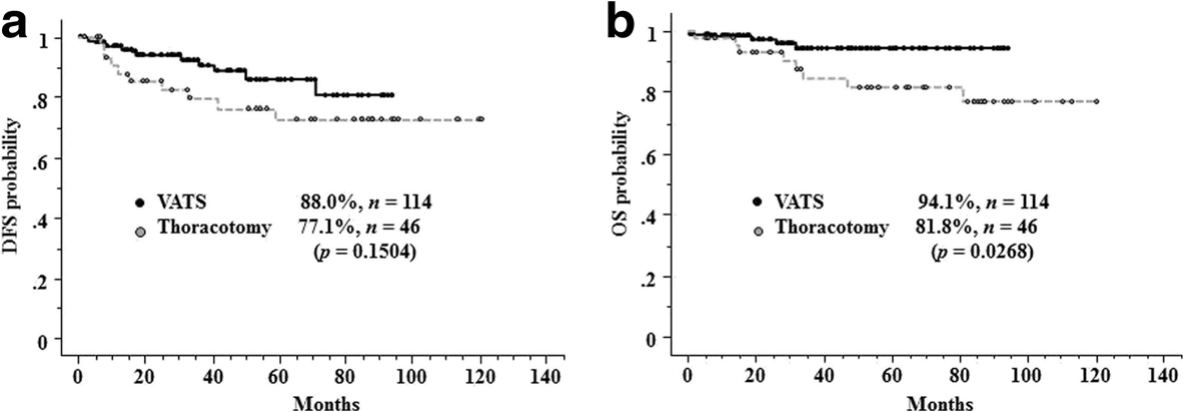
**Five-year disease-free survival (DFS) (a) and overall survival (OS) (b) for patients with clinical stage IA non-small-cell lung cancer (NSCLC).** The 5-year DFS rate was 88.0% in the VATS group and 77.1% in the thoracotomy group (*p* = 0.1504), and the 5-year OS rate was 94.1% in the VATS group and 81.8% in the thoracotomy group (*p* = 0.0268).

**Figure 2:**
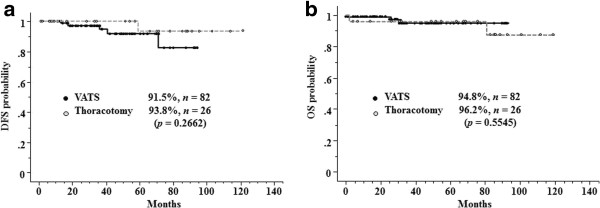
**Five-year DFS (a) and OS (b) for patients with pathological stage IA NSCLC.** The 5-year DFS rate was 91.5% in the VATS group and 93.8% in the thoracotomy group (*p* = 0.2662), and the 5-year OS rate was 94.8% in the VATS group and 96.2% in the thoracotomy group (*p* = 0.5545).

The median follow-up time was 44.8 months. The 5-year DFS rate was 88.0% in the VATS group and 77.1% in the thoracotomy group for clinical stage IA NSCLC (*p* = 0.1504, Figure 1a), and 91.5% in the VATS group and 93.8% in the thoracotomy group for pathological stage IA NSCLC (*p* = 0.2662, Figure 2a). The 5-year OS rate was 94.1% in the VATS group and 81.8% in the thoracotomy group for clinical stage IA NSCLC (*p* = 0.0268, Figure 1b), and 94.8% in the VATS group and 96.2% in the thoracotomy group for pathological stage IA NSCLC (*p* = 0.5545, Figure 2b).

### Accuracy of preoperative staging

The rate of accurate preoperative staging was 71.9% in the VATS group and 56.5% in the thoracotomy group (*p* = 0.2611). Inconsistencies between the clinical and pathological stages were mainly related to tumor size (12.3% and 17.4%), nodal metastasis (9.6% and 17.4%), and pleural invasion (14.9% and 19.6%) in the VATS and thoracotomy groups, respectively (Table 2).

**Table 2:** Inconsistencies between the clinical and pathological stages

	**Total (n = 160)**	**VATS (n = 114)**	**Thoracotomy (n = 46)**
Tumor diameter > 3.0 cm	20 (12.5%)	14 (12.3%)	8 (17.4%)
pNl or pN2	19 (11.9%)	11 (9.6%)	8 (17.4%)
Positive pl^a^	26 (16.3%)	17 (14.9%)	9 (19.6%)

^a^pl: pleural invasion.

### Recurrence

Recurrence occurred after the initial resection of 27 lesions in 22 patients (Table 3). Distant metastasis occurred after resection of 12 lesions (11 patients) in the VATS group, and eight lesions (5 patients) in the thoracotomy group. Interestingly, local recurrence occurred after resection of only one lesion in the VATS group and six lesions (5 patients) in the thoracotomy group (*p* = 0.0495). Univariate analyses showed that recurrence was associated with nodal metastasis (*p* < 0.0001 in the VATS group, *p* = 0.0136 in the thoracotomy group) and pleural invasion (*p* = 0.0174 in the VATS group, *p* = 0.0036 in the thoracotomy group). In the thoracotomy group, recurrence was also associated with lymphatic involvement (*p* = 0.0118) and vascular involvement (*p* = 0.0096) (Table 4). Multivariate analyses showed that recurrence was independently associated with nodal metastasis (*p* = 0.0026) in the VATS group, but was not independently associated with any factors in the thoracotomy group (Table 5).

**Table 3:** Sites of recurrence after surgery

	**VATS (13 lesions)**	**Thoracotomy (14 lesions)**
Carcinomatous pleuritis	1	4
Mediastinal LN^a^ metastasis	0	2
Local recurrence	1	6
Pulmonary metastasis	7	2
Brain metastasis	1	3
Bone metastasis	2	1
Hepatic metastasis	0	1
Others	2	1
Distant metastasis	12	8
**Total**	**13**	**14**

^a^LN: lymph node.

**Table 4:** Univariate analyses to identify factors associated with recurrence

		**VATS (n = l 14)**			**Thoracotomy (n = 46)**	
	**n**	**5-year DFS (%)**	**p**	**n**	**5-year DFS (%)**	**p**
Gender			0.3422			0.993
Male	50	84.7		33	78.7	
Female	64	87.2		13	73.6	
Pathology			0.8159			0.3595
Adenocarcinoma	91	84.3		32	71	
Squamous cell ca.	16	91.7		11	100	
Others	7	83.3		3	50	
Tumor diameter (cm)			0.5551			0.2765
≤3.0	100	88.2		40	75.7	
3.0<	14	68.8		6	58.4	
pN			<0.0001			0.0136
N0	103	90.1		38	84.7	
N1/N2	11	51.6		8	37.5	
Pleural involvement			0.0174			0.0036
Negative	97	90.3		37	87	
Positive	17	57.1		9	41.7	
Lymphovascular involvement			0.0536			0.0118
Negative	93	88.6		27	91.6	
Positive	21	73.8		19	53.7	
Vascular involvement			0.4972			0.0096
Negative	92	87.2		29	88	
Positive	22	79.7		17	58.1	

**Table 5:** Multivariate analyses to identify factors independently associated with recurrence in each group

**a. VATS group**			
**Variable**	**Hazard ratio**	**95% CI.**	**p**
pN0	015	0.043-0.516	0.0026
Negative pl^a^	0.389	0.111-1.369	0.1415
**b. Thoracotomy group**			
**Variable**	**Hazard ratio**	**95% CI.**	**p**
pN0	0.243	0.049-1.210	0.0841
Negative pl^a^	0.077	0.005-1.186	0.0986
Negative ly^b^	0.363	0.067-1.973	0.2407
Negative v^c^	0.893	0.138-5.764	0.9057

^a^pl: pleural invasion, ^b^ly: lymphatic involvement, ^c^v: vascular involvement.

## Discussion

VATS lobectomy is a minimally invasive technique for anatomic pulmonary resection. In patients with NSCLC, VATS lobectomy is associated with fewer complications and faster recovery than open thoracotomy [4,5,11-14] . In this study we also showed the significantly less bleeding in VATS group compared with thoracotomy group (Table 1). However, the most important parameter measuring the success of any oncologic surgical resection is long-term survival. The results of meta-analyses [13,14], randomized trials [4,5], and large retrospective series [11,12] indicate that this minimally invasive technique is safe and effective.

In this study, the 5-year OS and DFS rates in the VATS group were not significantly inferior to those in the thoracotomy group for clinical stage IA NSCLC (Figure 1), and OS was significantly better in the VATS group than in the thoracotomy group. Whilston et al. [13] reviewed 39 studies that compared VATS lobectomy with lobectomy via open thoracotomy. They found that patients who underwent VATS lobectomy had similar 1-, 2-, 3-, and 5-year survival rates compared with those who underwent open thoracotomy. Yan et al. [14] performed a similar systematic review and found that the 5-year survival rate was significantly higher in patients who underwent VATS lobectomy than those who underwent open thoracotomy for early-stage NSCLC (VATS relative risk, 0.72; *p* = 0.04).

The significantly better OS in the VATS group reflects the inaccurate preoperative diagnosis of clinical stage IA NSCLC in some patients. Preoperative staging was based on the findings of imaging examinations such as CT and F18-fluorodeoxyglucose positron emission tomography. The rate of accurate diagnosis of clinical stage IA NSCLC was 71.9% in the VATS group and 56.5% in the thoracotomy group (*p* = 0.2611). In both groups, inconsistencies between the clinical and pathological stages were mainly related to lymph node status, pleural involvement, and tumor size (Table 2). Stage migration occurs when there is a difference between the clinical and pathological T, N, or M stage, which occurs in up to 53% of patients who undergo resection of lung cancer [15]. To improve the accuracy of preoperative staging, imaging examination findings should be more precisely evaluated, especially tumor size and lymph node status. However, pleural involvement cannot be determined preoperatively, as this is diagnosed by pathological examination, even if preoperative CT shows pleural indentation. It is therefore important to develop more effective methods of determining pleural invasion on preoperative imaging examinations. It should also be kept in mind that selection bias influences the rate of accurate diagnosis of clinical stage IA NSCLC. For example, VATS lobectomy is considered preferable in female patients with small adenocarcinomas, whereas male patients with squamous cell carcinoma are less likely to be selected for VATS lobectomy because they may have emphysematous lungs and non-specific lymph node enlargement due to heavy smoking or their working history, which may increase the difficulty of surgery. During the early period of performing VATS lobectomy, we may have avoided preforming VATS in the latter group of patients. The significant differences in sex distribution, lymphatic involvement, and vascular involvement between the VATS and thoracotomy groups may reflect such a bias (Table 1). Furthermore, the much higher rate of nodal upstaging in the thoracotomy group than in the VATS group may reflect dissection of a lower number of lymph nodes, or inadequate dissection of lymph nodes, during VATS lobectomy compared with open thoracotomy in patients with clinical stage IA NSCLC. However, we did not evaluate the number of dissected lymph nodes in each group. Merritt et al. [16] reported that lymph node dissection may be inadequate during VATS lobectomy. Boffa et al. [17] also showed the incompleteness of the peribronchial and hilar node evaluation in the VATS group. In this study, upstaging from N0 to N1 or N2 was more common in the thoracotomy group (17.4% versus 9.6%, p=0.1706) as with previous reports [17]. However, this study found that local control was better in the VATS group, and the prognosis of patients with pathological stage I NSCLC was not significantly different between the VATS and thoracotomy groups. These results suggest that VATS lobectomy is a feasible treatment for pathological stage IA NSCLC and indicate that differences in nodal upstaging result from patient selection. However, this study found that local control was better in the VATS group, and the prognosis of patients with pathological stage I NSCLC was not significantly different between the VATS and thoracotomy groups. These results suggest that VATS lobectomy is a feasible treatment for pathological stage IA NSCLC. Multivariate analysis indicated that the accuracy of preoperative staging is the most important factor needed to improve the prognosis of VATS lobectomy for clinical stage IA NSCLC.

Some centers have reported that they perform all pulmonary surgical procedures by VATS, including pulmonary artery reconstruction and bronchoplasty [18,19]. Our center currently uses VATS lobectomy only for clinical stage IA and IB NSCLC, but we may be able to extend the indications for this procedure to more advanced cases of NCSLC. The long-term outcomes after VATS lobectomy still require further evaluation. The present study is limited by its retrospective nature, and a larger prospective randomized study is required to reach definitive conclusions regarding the efficacy of VATS lobectomy for the treatment of NSCLC.

## Conclusions

The DFS and OS were not inferior after VATS compared with thoracotomy in clinical stage I NSCLC. Local control was significantly better after VATS than after thoracotomy. However, preoperative staging lacked sufficient accuracy.

## Abbreviations

VATS: Video-assisted thoracic surgery; NSCLC: Non-small cell lung cancer; DFS: Disease free survival; OS: Overall survival; CT: Computed tomography; MRI: Magnetic resonance imaging; FDG-PET: F18-fluorodeoxyglucose positron emission tomography; LN: Lymph node; Pl: Pleural invasion; Ly: Lymphatic involvement; V: Vascular involvement.

## Competing interests

The authors declare that they have no competing interest.

## Authors’ contributions

MH contributed to the drafting of this manuscript, data collection and statistical analysis, and RK, AK, HS and MG contributed to the study design and statistical analysis. HY and AY contributed to data collection. All authors have read and approved of the submission of the final manuscript.
